# *Coxiella burnetii* Seroprevalence in Small Ruminants in The Gambia

**DOI:** 10.1371/journal.pone.0085424

**Published:** 2014-01-15

**Authors:** Marieke Klaasen, Hendrik-Jan Roest, Wim van der Hoek, Bart Goossens, Arss Secka, Arjan Stegeman

**Affiliations:** 1 Department of Farm Animal Health, Faculty of Veterinary Medicine, Utrecht University, Utrecht, The Netherlands; 2 Department Bacteriology and TSEs, Central Veterinary Institute, part of Wageningen University and Research Centre, Lelystad, The Netherlands; 3 Epidemiology and Surveillance Unit, National Institute for Public Health and Environment, Bilthoven, The Netherlands; 4 SOS Children’s Villages International, Bakau, The Gambia; 5 International Trypanotolerance Centre, Banjul, The Gambia; Texas A&M Health Science Center, United States of America

## Abstract

**Background:**

Q fever is a zoonosis caused by *Coxiella burnetii*, a Gram negative bacterium present worldwide. Small ruminants are considered the main reservoirs for infection of humans. This study aimed to estimate the extent of *C. burnetii* infection among sheep and goats in part of The Gambia.

**Methodology/Principal Findings:**

This survey was carried out from March to May 2012 at two areas in The Gambia. The first area comprised a cluster of seven rural villages situated 5–15 km west of Farafenni as well as the local abattoir. A second sampling was done at the central abattoir in Abuko (30 km from the capital, Banjul) in the Western Region. Serum samples were obtained from 490 goats and 398 sheep. In addition, 67 milk samples were obtained from lactating dams. Sera were tested with a Q fever ELISA kit. *C. burnetii* DNA was extracted from milk samples and then detected using a specific quantitative multiplex PCR assay, targeting the IS*1111a* element. A multivariable mixed logistic regression model was used to examine the relationship between seropositivity and explanatory variables. An overall seroprevalence of 21.6% was found. Goats had a significantly higher seroprevalence than sheep, respectively 24.2% and 18.5%. Seropositive animals were significantly older than seronegative animals. Animals from the villages had a significantly lower seroprevalence than animals from the central abattoir (15.1% versus 29.1%). *C. burnetii* DNA was detected in 2 out of 67 milk samples, whereas 8 samples gave a doubtful result.

**Conclusion/Significance:**

A substantial *C. burnetii* seroprevalence in sheep and goats in The Gambia was demonstrated. People living in close proximity to small ruminants are exposed to *C. burnetii*. Q fever should be considered as a possible cause of acute febrile illness in humans in The Gambia. Future studies should include a simultaneous assessment of veterinary and human serology, and include aetiology of febrile illness in local clinics.

## Introduction

Malaria has long been presumed to be the overwhelming cause of febrile illness in humans in sub-Saharan Africa. However, during the last decade a substantial decline in malaria prevalence and incidence has been observed in African countries [1]–[3]. For example, in The Gambia, during the 2008 malaria season, only 11% (24/224) of febrile episodes detected during 22 weeks follow up of a cohort of 800 children in Farafenni area were due to malaria [4]. Consequently, miss-diagnosis of presumed clinical malaria might now be present, which threatens the sustainability of currently effective antimalarial treatment, while treatable bacterial diseases that cause febrile illness are likely to be missed [2], [5]. The few studies that have been done show that these treatable bacterial diseases are often emerging or neglected zoonoses, such as Q fever [5], brucellosis [6], African tick-bite fever [7] and leptospirosis [8]. It is therefore important to get better insight into the extent of the problem of these zoonoses in the human and animal populations of sub Saharan Africa.

Q fever is a zoonosis caused by *Coxiella burnetii*, a Gram negative bacterium present worldwide [9]. In humans an infection with *C. burnetii* either goes by unnoticed or presents itself as a flu-like illness, as pneumonia, or as hepatitis. However, in 1 to 5% of cases, the disease progresses to a chronic stage characterized mainly by endocarditis or vascular infection [10], [11].

A recent study in Tanzania among hospitalized febrile patients showed that 13.5% had acute Q fever or Rickettsial infection [5]. Considering Q fever alone, the seroprevalence in apparently healthy people varies between 1% to 37% in different sub-Saharan countries [12]–[15]. In The Gambia, baseline sera from the cohort of 800 children mentioned above, were tested for antibodies against *C. burnetii* by ELISA, resulting in a seroprevalence of 8.3% [19].

Reservoirs of *C. burnetii* include many wild and domesticated mammals, birds and ticks [10]. *C. burnetii* is transmitted between domesticated animals such as sheep, goats, cattle, cats, and dogs, either by tick bite or through contact with infected excreta [9]. In animals, *C. burnetii* infection does not usually provoke severe symptoms. However, in cattle it has been associated with infertility and in small ruminants (goats and sheep) the infection can result in late abortions [16]. The massive shedding of *C. burnetii* during such abortions makes sheep and goats the main reservoirs responsible for infection of humans [9], [16]. Analysis of recent human Q fever outbreaks showed that these outbreaks are associated with small ruminants rather than cattle [18]. Infection of humans is primarily via inhalation of contaminated aerosols [10]. The association between high seroprevalence in humans and the prominent role of livestock breeding in many parts of sub-Saharan Africa has been suggested in several studies [13]–[14], [17]. However, as far as the authors have been able to establish, no data are available to prove this suggestion.

The objective of this study was to estimate the prevalence of *C. burnetii* infection among small ruminants in Farfenni area of The Gambia. This location was chosen because the *Coxiella burnetii* seroprevalence in children had been examined in this area in an earlier study [19].

## Methods

### Study Sites

**Figure 1:**
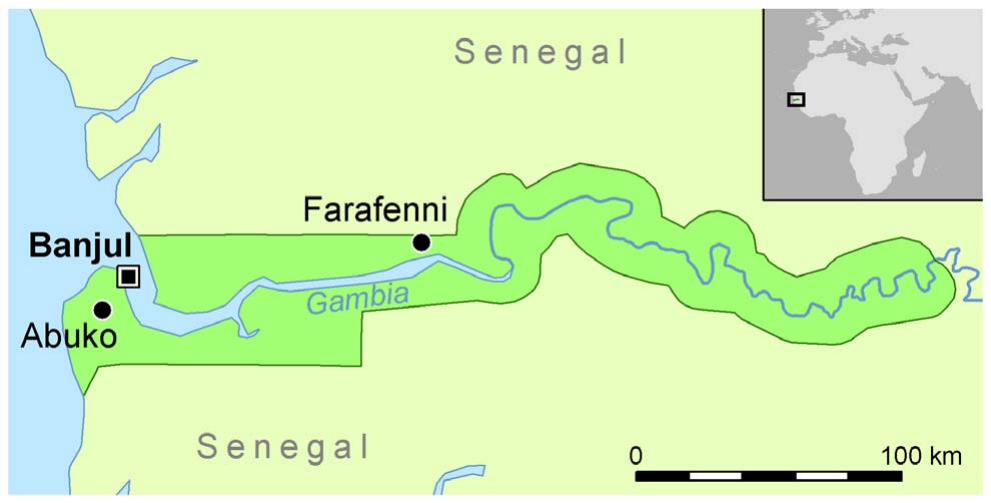
Geographical location of Farafenni area, Abuko and Banjul (capital city) within The Gambia, Western Africa.

This survey was carried out from March to May 2012. In these months it is expected that the infection status of the small ruminants is stabilized, following the period of August to November during which most parturitions occur. The sampling was performed at two areas comprising different sites in The Gambia. The first area is located around Farafenni, a town situated on the Trans-Gambia Highway in the North Bank Region, just south of the border with Senegal (Figure 1). It is an important market town with a high number of travellers from Senegal. The area comprised a cluster of seven rural villages (Alkali Kunda, Jarjary, India, Yallal, Daru Yallal, Jumansareba and Conteh Kunda Nicci) situated 5–15 km west of Farafenni, as well as the local Farafenni abattoir. The villages are the same where human samples for a malaria cohort study were collected [4] and which were also used for the human Q fever survey [19].

A second sampling was done at the central abattoir in Abuko (30 km from the capital, Banjul) in the Western Region. The Animal Health and Production Services (AHPS) of The Gambia estimated 1000 sheep and 1500 goats to be living in the Farafenni area. According to AHPS records, 10 to 15 small ruminants are slaughtered daily at the local abattoir in Farafenni, whereas the central abattoir in Abuko slaughters on average 50 small ruminants a day.

### Study Population

The traditional and still widely practiced livestock system in The Gambia is agropastoralism, a low-input mixed crop-livestock system with extensive grazing and a low level of integration [20]. In this type of production system livestock are dependent on natural forage and leftovers of the cropping season [20]. During the dry season sheep and goats are left free for grazing whereas in the rainy season (cropping season) the sheep and goats are either tethered in the vicinity of the village or herded so as to avoid crop damage. Most of the rural households own a few small ruminants which serve as savings or emergency cash (e.g. to pay for funerals or school fees), provide protein (meat or milk) or non-food commodities (manure, hides) and are used in religious celebrations [21]. The indigenous small ruminant breeds are the Djallonké sheep and the West African Dwarf (WAD) goats. With a height range of 40–60 cm for sheep and 30–50 cm for goats, these breeds are classified as dwarf breeds [22]. Sahelian long-legged sheep and goats from Senegal and Bali Bali sheep from Mali are however more and more often imported specially at certain religious occasions such as Tobaski (Eid al-Adha) [21]–[22].

### Study Design

Preceding the sampling, introductory meetings were held in the seven villages with the community elders and owners, and at both abattoirs with the health inspectors and slaughterers. All owners gave consent for their animals to be sampled for the study. In each of the seven villages 25 sheep and 25 goats were randomly selected for sampling. At both abattoirs 156 sheep and 156 goats offered for slaughtering were targeted for sampling. This sample size, calculated using WinEpiscope 2.0 [23], was expected to enable detection of a 30% difference in seropositivity between different subpopulations, for instance sheep versus goats or young versus old animals. Assuming a statistically worst-case scenario prevalence of 50% and a confidence interval of 95%, calculations with an absolute precision of 7,85% can be realized.

### Ethics Statement

The study described in this manuscript was conducted in compliance with legislation on animal experimentation and practicing veterinary medicine of both The Netherlands and The Gambia. The study is not an animal experiment, but an epidemiological study in the field using common sampling methods for routine diagnostic purposes. According to Dutch legislation, such studies do not need approval from an animal ethics committee, but they need to be performed according to the Dutch Veterinary Practice Act.

### Sampling

A structured questionnaire was used to record data including the name of the owner (villages) or trader (abattoirs), species, breed, sex, estimated age, lactating or not, and, if lactating, the suckling lamb(s) or kid(s) were also sampled and their relationships were documented. In the villages milk samples were taken from the lactating dams which were included in the serum sampling. Age was estimated by dentition as defined earlier [21].

Blood samples were collected from the jugular vein in evacuated blood collecting tubes of 8 ml (Greiner Bio-One, Kremsmünster, Austria), using 20 G×1.5 Multi-Sample Blood Collection needles (Greiner Bio-One, Kremsmünster, Austria). The tubes were left at ambient temperature for circa 1 hour and then stored in a cool box on ice and/or in a refrigerator. Samples were centrifuged within 18 hours (2500 g,10 min) and serum samples were then stored frozen.

After cleaning the teats with disinfectant wipes and forestripping, milk samples were collected in 15 ml polystyrene milk tubes (Greiner Bio-One, Kremsmünster, Austria). Milk samples were preserved with Broad Spectrum Microtabs II (D&F Control Systems, Norwoord, USA), containing bronopol and natamycin. The milk samples were stored frozen until testing.

### Diagnostic Tests

Sera were tested with the Q fever LSI ELISA kit (LSI, Lissieu, France) [24], according to manufacturer’s instructions. In addition to the negative and positive control samples supplied by the ELISA kit, an extra positive control sample was added to every plate. This extra control was adjusted to a titer just above the cut off value of the test. The results were expressed as optical density Sample/Positive control (S/P) ratio, corrected for the negative control. A serum sample was considered to be positive when the S/P ratio of the serum was >40 and seronegative ≤40.

DNA was extracted from the milk using the NucliSens easyMag extractor (bioMérieux, Marcy l’Etoile, France) according to the manufacturer’s recommendations. In case of repeated errors, DNA extraction was performed using a DNA tissue kit (DNeasy Blood and Tissue kit; QIAGEN, Hilden, Germany) according to the manufacturer’s guidelines. *C. burnetii* DNA was detected using a specific quantitative multiplex PCR assay, targeting the IS*1111a* element. Positive, negative and inhibition controls were included, as described earlier [25]. A milk sample was considered positive when the cycle threshold (Ct) value was ≤36, negative when the Ct was ≥40 and doubtful when the Ct was between >36 and <40. The source for the thresholds is the repeatability in goat milk: below the lower threshold (positive result) the repeatability is 100%; above the threshold and the negative result (doubtful result) the repeatability is decreasing towards zero.

### Statistical Analyses

A multivariable mixed logistic regression model was used to examine the relationship between seropositivity and explanatory variables ‘species’, ‘breed’, ‘sex’, ‘age’ and ‘location’. The latter included as a random effect [26], as animals from the same study site might not be independent of each other. Most variables, including the dependent variable, were dichotomous. Age was classified in three groups: <1 year, 1–3 years and ≥4 years. Location was categorized based on the three study sites: villages Farafenni area, Farafenni abattoir and Abuko abattoir.

A backward stepwise selection on the full model was performed to find the best fitting model to describe the dataset. Selection of the best fitting model was based on the value of Akaike’s Information Criterion (AIC). The model with the lowest AIC value was considered the best fitting model, with the AIC being the –2 * loglikelihood +2 * the number of parameters in the model. The odds ratio and 95% confidence interval for the explanatory variables were calculated. The analyses were performed using R software, version 2.12.2 [27] and package lme4, version 0.999375–42 [28] for generalized linear mixed-effects models using the Laplace approximation method.

## Results

### Descriptive Statistics

Serum samples were obtained from 490 goats and 398 sheep (Table 1). During the four weeks of sampling at Farafenni abattoir, the number of sheep offered for slaughter was limited to 66 sheep.

**Table 1:** Number of serum samples at the different study sites.

	Goats	Sheep
Villages Farafenni area	175	175
Farafenni abattoir	156	66
Abuko abattoir	159	157

**Figure 2:**
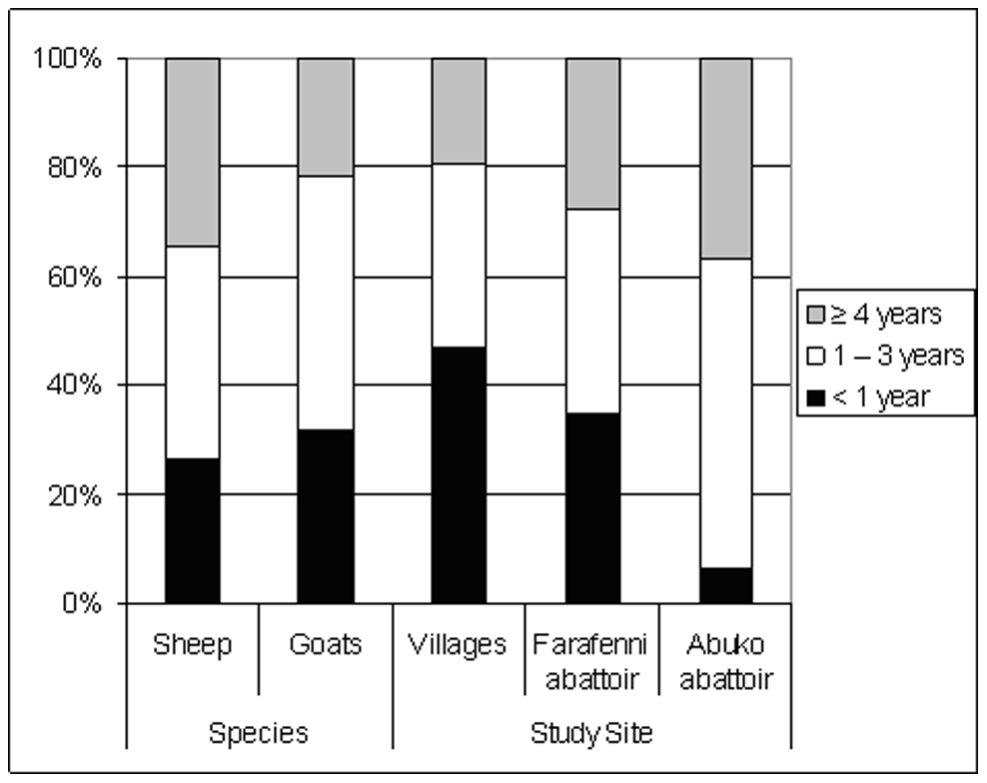
Proportion of sampled animals per age group, related to species and study site.

As to the sex distribution: 68.6% of the goats and 80.4% of the sheep were female. Practically all animals sampled in the villages belonged to the indigenous WAD goats and Djallonké sheep, whereas at Abuko abattoir 81.1% of the animals were exotic breeds. At Farafenni abattoir the proportion indigenous to imported breeds was 55.7% to 44.3%. Of the animals sampled in the villages 47% was younger than 1 year of age. At Abuko abattoir the middle and the oldest age groups were most prominent (Figure 2).

Milk samples were collected from lactating dams in the villages only. In total 67 milk samples from lactating dams (33 goats and 34 sheep) were collected, in conjunction with serum sampling. In each of the seven villages between 9 to 12 milk samples were collected, except for Jarjary, where only 3 milk samples could be obtained.

### Seroprevalence and Associated Factors

The general linear model was performed based on 879 complete records, as 9 records contained missing values. Based on the AIC, the best fitting model included explanatory variables location, age and species. The degree of clustering within the 9 different locations (two abattoirs and seven villages) was very small in the general mixed model with a variance of 2.3927e-14. In the best fitting model the correlation was negligible (σ^2^ = 0). The best fitting model showed no significant difference in the odds of seropositivity in animals being offered for slaughtering at Farafenni abattoir or at Abuko abattoir (OR 0.78, p = 0.26) (Table 2).

**Table 2:** Risk of being anti-*Coxiella burnetii* antibody seropositive associated with animal characteristics and demographics, multivariable mixed logistic regression.

Variable		Number of animals	Seroprevalence (%)			
				OR	95% CI	p-value
Total		879	21.6			
Location	Abuko abattoir	313	29.1	1.0 (ref.)		
	Farafenni abattoir	221	21.3	0.78	0.51–1.20	0.26
	Villages	345	15.1	0.61	0.40–0.91	0.02
Estimated age group	<1 year	256	9.8	1.0 (ref.)		
	1–3 years	379	26.1	2.78	1.69–4.56	5.46e-05
	≥4 years	244	27.0	3.10	1.83–5.25	2.67e-05
Species	Goat	484	24.2	1.0 (ref.)		
	Sheep	395	18.5	0.65	0.46–0.92	0.02

OR, Odds ratio; CI, confidence interval.

Animals located in the villages on the other hand had a significantlylower seroprevalence than animals offered for slaughtering at Abuko abattoir (OR 0.61, p = 0.02). Seropositive animals were significantly older than seronegative animals (Table 2). Sheep had a significantly lower risk of being seropositive as compared to goats (OR 0.65, p = 0.02).

### Milk Samples; PCR Analysis and Relation to Seroprevalence Results

*C. burnetii* DNA was detected in 2 out of 67 milk samples, whereas 8 samples gave a doubtful (i.e. Ct values >36 and ≤40) result. In 57 samples no *Coxiella* DNA was found. One of the dams with a positive milk sample was seropositive; three dams with a doubtful PCR result also were seropositive.

Of the 67 lactating dams, 19 (28.4%) were seropositive and 48 (71.6%) were seronegative. The serum of 14 kids of the seropositive lactating dams and 39 kids of the lactating seronegative dams were included in the sampling. These kids were all less than 12 months of age, with a minimum of one week of age. From the seropositive lactating dams two kids (14.3%) were seropositive and of the seronegative lactating dams no kids were seropositive. This difference is not significant (Fisher’s exact test, p = 0.081).

## Discussion

In this study in The Gambia, 18.5% of sheep and 24.2% of goats had antibodies to *C. burnetii*. To our knowledge, this is the first seroprevalence study of *C. burnetii* in sheep and goats in West Africa. A recent literature review on the prevalence of *C. burnetii* infection in domestic ruminants in different countries worldwide, revealed a wide variation in reported prevalence and study quality [17]. The overall mean prevalences on animal level were 15% and 27% for sheep and goats respectively [17]. Out of the 69 publications reviewed, only four studies on small ruminants were performed in African countries [17]. In a well-designed study in Chad, Central Africa, seroprevalences of 11% and 13% were found in sheep and goats respectively [29]. Results of our study showed a higher seroprevalence among small ruminants, which suggests that Q fever is of considerable importance in the small ruminant population in The Gambia.

The age of the animals appeared to be the most significant risk factor for seropositivity. The older the animals are, the higher the risk of being seropositive. This difference was especially notable between animals younger than 1 year of age versus older animals. Animals between 1 to 3 years of age and animals of 4 years or older were shown to be respectively 2.8 and 3.1 times more likely to be seropositive as compared to animals younger than 1 year of age. These findings are indicative of horizontal rather than vertical transmission. *C. burnetii* infections usually induce an immune response which provides long-lasting protection against further disease [9]. However, the risk of being seropositive seems to reach a plateau, after which seroprevalence decreases slightly with age.

Taking into account all other variables in the model, the odds of being seropositive for animals located at the villages was 0.6 (lower risk) as compared to animals being offered for slaughtering at Abuko abattoir. At Abuko abattoir animals from all over the country and abroad are assembled [21]. As such, these animals might originate from entirely different populations with different prevalence figures. In contrast with Farafenni abattoir, where animals are slaughtered directly, most animals arriving at Abuko are kept at the adjoining marketplace preceding slaughtering. Animals at Abuko are sometimes kept several months before being slaughtered [21]. In addition, to what extent these animals are a random selection of these populations is unknown, as selection criteria used by livestock keepers to sell a specific animal are not identified. However, the majority of animals offered for slaughtering appeared to be clinically healthy, which matches with earlier observations [21]. As such, a difference in origin, (extensive) travelling and assembling, and a prolonged stay at the adjoining marketplace, are possible risk factors for the higher *C. burnetii* seroprevalence found in sheep and goats offered for slaughtering at Abuko abattoir.

Furthermore, in the present study sheep appeared to have a significantly lower risk of being seropositive as compared to goats. Differences in intrinsic susceptibility to *C. burnetii* between sheep and goats have not been described in the literature. Some studies found higher seroprevalences in sheep [30]–[32], whereas others found higher seroprevalences in goats [29]. Although the LSI ELISA kit uses antigen of an ovine strain, due to the use of a monoclonal anti-protein G HRP labeled conjugate, the test is equally suitable for bovine, ovine and caprine species. An in-house validation of the test by the Central Veterinary Institute (The Netherlands) found similar results in known positive and negative sera from both sheep and goats (unpublished results). As such, transmission within the Gambian sheep population seems to be different from that in the goat population, and there probably is no random mixing between the species.

Most small ruminants in The Gambia are kept in free-roaming village-based flocks with only limited management or capital inputs [33]. During the dry seasons, most of the animals roam freely, but sheep stay closer to the villages than goats [34]. In addition, village-based sheep in The Gambia are more often tethered at night as compared to village-based goats [34]. On the other hand, in case goats are tethered at night, their night shelters are more often provided with a roof and wall as compared to the shelters of sheep [34]. In terms of exchange for cash or kind, goats are considered the cheapest form of trade followed by sheep, donkeys, cattle and finally, horses [33]. In addition, sheep are preferred for slaughter when celebrating religious festivities or ceremonies, whereas goats are preferred for commercial slaughter and the preparation of afra (grilled meat) [21]. This explains the limited number of sheep offered for slaughtering at the local abattoir in Farafenni during the sampling period. In addition, the differences in trade value and accompanying management practices might explain the difference in seropositivity between the two species.

In a small proportion of lactating dams *C. burnetii* DNA was found in the milk, while a larger proportion of milk samples gave a doubtful result. This indicates that only low *Coxiella* DNA loads were detected. Shedding prevalence is an indicator of current infection and can be used as a measure to estimate the risk of transmission between ruminants and from ruminants to humans [17]. However, shedding of *C. burnetii* through milk may be continuous or intermittent [9]. Additionally, some animals seroconvert without detectable shedding, whereas other shedding animals may never seroconvert [35], although this has been questioned and might be explained by cross contamination for the environment [36].

*C. burnetii* can survive in the open for weeks and is highly infectious [10]. A clear relationship between dry weather, strong wind, and the spread of infection in dust from a variety of animal sources has been reported [9]. In addition, areas with low vegetation densities and low groundwater levels are more prone to transmission over large areas [37]. The climate in The Gambia is semi-arid with distinct dry (December to June) and wet (July to November) seasons [21]. In an earlier study it was found that the number of parturitions in sheep and goats in The Gambia was higher between August and November as compared to other months [34]. As ruminants have been reported to shed large amounts of bacteria during parturition [9], [16] and as the bacterium is highly resistant [10], the onset of the dry season, which is accompanied by strong winds, can be considered as a high risk period. Hence, an incident resulting in shedding of large numbers of bacteria may quickly impact a large area.

In the human survey, based on sera collected for a malaria study in 2008, the average seroprevalence in the same seven villages found was 8.8%, ranging from 5.1 to 14.3% [19]. Both the veterinary and human data show significant differences in seroprevalence between the villages. Comparing the human and veterinary seroprevalence data, the results show a poor correlation. Direct relationships between the seroprevalence in the children and the small ruminants in the Farafenni villages could not be demonstrated, because demographic information was lacking and because of the four-year time difference between the collection of the human and the veterinary data. However, given the fact that antibodies to *C. burnetii* are present in significant numbers in both humans and animals, possible linkage should be investigated further. In order to determine to what extent *C. burnetii* prevalence rates in humans and animals interact, more data will have to be collected simultaneously on both humans and animals in different geographical locations. Furthermore, more research could be carried out in villages with both high and low prevalence rates in humans and animals, in order to determine what types of human behavior or interaction with animals might cause higher incidence levels.

This serological survey of *C. burnetii* seroprevalence in small ruminants in The Gambia demonstrates a considerable prevalence of current or past infection in the sheep and goat population. The species and age of the animals as well as their location and origin are of influence on the seropositivity of *C. burnetii*. Although a direct link between the human and veterinary data could not be demonstrated, there are clear zoonotic implications. *C. burnetii* is highly contagious and very resistant in the environment. People living in a Q fever endemic area are at risk of getting infected. To avoid incorrect diagnoses of febrile illnesses as being malaria, resulting in an overuse of anti-malarial drugs, it is important that other prevalent (treatable) causes are considered and can be diagnosed. As such, Q fever is one of the diseases that should be taken into account. While malaria rapid diagnostic tests are now widely available, there is a need for low-cost point-of-care diagnostics for non-malarial febrile illness. Simultaneous assessments of veterinary and human serology with aetiologic studies of febrile illness in local clinics would provide important information of the importance of zoonotic pathogens such as *C. burnetii*.
